# Using the axillary reverse mapping technique to screen breast cancer patients with a high risk of lymphedema

**DOI:** 10.1186/s12957-020-01886-9

**Published:** 2020-06-01

**Authors:** Siyao Liu, Nan Wang, Ping Gao, Peng Liu, Houpu Yang, Fei Xie, Siyuan Wang, Miao Liu, Shu Wang

**Affiliations:** 1 Breast Center, Peking University People’s Hospital, Beijing, China; 2 Tianjin Medical University Cancer Institute and Hospital, Tianjin, China; 3 Nuclear Medicine Department, Peking University People’s Hospital, Beijing, China

**Keywords:** Lymphedema, Axillary reverse mapping (ARM), Breast cancer

## Abstract

**Background:**

Preventing breast cancer-related lymphedema (BCRL) by preserving upper lymphatic drainage is still controversial. Our study aimed to use the axillary reverse mapping (ARM) technique in patients who underwent axillary surgery to analyse the correlation between postoperative residual ARM nodes and the occurrence of lymphedema, select candidates at high risk of developing lymphedema, and analyse the oncologic safety of ARM nodes.

**Methods:**

Patients undergoing sentinel lymph node biopsy (SLNB) or axillary lymph node dissection (ALND) from October 2015 to February 2016 at the Peking University People’s Hospital Breast Center were prospectively recruited for the study. ARM was performed in all patients before surgery. ARM nodes were separated from SLNB and ALND specimens. Data were collected on the identification of ARM nodes before surgery, number of residual ARM nodes after surgery, nodal status, crossover rate, and correlation between residual ARM nodes and the occurrence of lymphedema.

**Results:**

The analysis included 78 patients. Of the 53 patients who underwent SLNB, crossover between ARM nodes and breast sentinel lymph nodes (SLNs) was seen in 22 specimens. The incidence of ARM node metastasis was 1.89% (1/53) and 25% (9/36) in the SLNB and ALND groups, respectively. The number of positive axillary lymph nodes was associated with the involvement of ARM nodes (*P* = 0.036). The incidence of residual ARM nodes was significantly higher in the SLNB group than in the ALND group (67.6% vs. 37.9%, *P* = 0.016). The incidence of lymphedema was significantly higher in the ALND group than in the SLNB group (33.3% vs. 5.4%, *P* = 0.003).

**Conclusions:**

For SLNB, the ARM technique can help to resect SLNs more accurately. However, we do not recommend using the ARM technique to preserve ARM nodes in patients undergoing ALND due to oncologic safety issues. Nevertheless, we can predict patients undergoing axillary surgery who are more likely to have a high risk of lymphedema by assessing residual ARM nodes.

**Trial registration:**

This study was registered on ClinicalTrials.gov in February 2016.

The clinical trial registration number is NCT02691624.

## Introduction

Lymphedema (LE) following axillary surgery continues to be a major long-term complication of women who survive breast cancer. As previously reported, axillary lymph node dissection (ALND) and sentinel lymph node biopsy (SLNB) are associated with a 7–77% [1] and 3–13% risk of lymphedema (LE) [2], respectively. The mechanism of lymphedema is not clear. It has been hypothesized that the main reason for lymphedema after ALND or SLNB is the disruption of upper lymphatic drainage. The axillary reverse mapping (ARM) technique can possibly decrease lymphedema by identifying and preserving upper lymphatic drainage during ALND and SLNB [3]. In 2007, Thompson and Nos [4, 5] first reported that the ARM technique could reduce the incidence of postoperative upper extremity lymphedema if the ARM nodes, identified by the ARM technique, were not removed. Since then, several studies have also demonstrated similar results [6–8]. However, another study showed no decrease in the rate of lymphedema by preserving ARM nodes [9–11]. Furthermore, some studies had conflicting findings regarding the oncologic safety of the ARM technique [5, 12, 13], which has limited routine clinical use in long-term follow-up.

Although some issues concerning the ARM technique have been raised, it remains a useful method to distinguish upper extremity lymph nodes from those of the breast lymphatic drainage system. In this study, to explore the mechanism of lymphedema and identify patients at high risk for lymphedema, we applied the ARM technique in patients who underwent axillary surgery to analyse the correlation between postoperative residual ARM nodes and the incidence of lymphedema as well as the oncologic safety of ARM nodes.

## Methods

### Patients

This study was a prospective clinical trial conducted at the Peking University People’s Hospital (PKUPH) and was registered on ClinicalTrials.gov. The clinical trial registration number was NCT02691624. Approval from the ethics committee of PKUPH was obtained before patient recruitment. Female patients diagnosed with breast cancer who underwent either SLNB or ALND were enrolled from October 2015 to February 2016. Patients with distant metastases, inflammatory breast cancer, previous axillary surgery, or hypersensitivity to dextran or indocyanine green (ICG) were excluded from the study. Written consent was obtained from all of the patients.

### Axillary surgery

#### The sentinel lymph node biopsy procedure

The SLNB procedure was performed with a dual mapping technique involving the injection of methylene and ICG. SLNs were detected following the migration of the blue dye and ICG through the lymphatics. Blue-stained and/or fluorescent-positive lymph nodes were found and removed. The excised SLNs were cut longitudinally into two halves; half were frozen for immediate examination, and the other half were embedded in paraffin, sectioned, and examined with microscopy after haematoxylin and eosin (H&E) staining and immunohistochemical (IHC) staining [14, 15].

#### The axillary lymph node dissection procedure

In patients who underwent a mastectomy, ALND was performed if SLNs were positive. In patients who underwent breast-conserving therapy, a second ALND procedure was performed according to the Z0011 criteria [2]. ALND was defined as an anatomic level I and II dissection on the affected side.

### The axillary reverse mapping procedure

The patients received intradermal injections of 99mTc-DX (0.1–0.15 ml) on the back of the hand ipsilateral to the tumour 2 h before surgery [16, 17]. One hour after the injections, single-photon emission computed tomography (SPECT/CT) was performed to identify the draining lymph nodes in the axillary and clavicular regions [8]. When SLNB and/or ALND were performed, the ARM nodes were not specifically preserved. After axillary specimens were removed, we used a γ-detector to detect the presence of hot lymph nodes as well as ARM nodes in the SLNB or ALND specimens and separated them. Then, the separated ARM nodes were sent for processing and histopathologic review. After the SLNB and/or ALND procedures were performed, a γ-detector was used again to detect whether hot lymph nodes were left in the axillary and clavicular region immediately after the surgeries were completed. All surgeries were performed by the same surgeon.

### Lymphedema assessment and follow-up visits

All patients completed a specific follow-up visit to check for the occurrence of any breast cancer-related lymphedema (BCRL). In our study, arm circumference was measured at ten sites on both limbs using a tape-line measurement. The locations measured were mid-metacarpal, the wrist, at 5 and 10 cm above the wrist, elbow fold, at 10 and 5 cm below the elbow fold, and at 15, 10, and 5 cm above the elbow fold. All measurements were performed by the same investigator. Lymphedema was defined as a 2-cm or greater increase in ipsilateral arm measurements compared with contralateral arm measurements at any of the 10 measured locations on the limb [2]. In addition, patients were asked to indicate whether there was swelling during a standardized interview by answering the following question with a “yes” or “no”: in the past year, have you experienced swelling of the upper extremity on the breast cancer-treated side? Self-assessment was described as negative or positive for BCRL [18]. Follow-up visits were scheduled at 3, 6, 9, and 12 months postoperatively for the first year and then every half year thereafter. The median follow-up period was 36.3 (range, 25.1–40.0) months. Patients with bilateral breast cancer were not included in the lymphedema analysis.

### Data collection and statistics

All clinical data were analysed using SPSS (IBM Corp., Armonk, NY) 24.0 software. Descriptive analyses were used to analyse the number and metastatic status of the ARM nodes, the crossover between ARM nodes and SLNs, and the incidence of lymphedema. When analysing the factors that influence the presence of ARM node metastases in patients who received ALND, the chi-square test or Fisher’s exact test was used to analyse the categorical variables, and the rank-sum test was used to analyse the continuous variables. The relationship between the incidence of lymphedema and ARM node data was calculated by using the chi-square test or Fisher’s exact test. All *P* values were 2-tailed, and *P* < 0.05 was considered statistically significant.

## Results

### Patients

Between October 2015 and February 2016, a total of 78 patients who underwent SLNB and/or ALND were included in the study. Six patients were lost to follow-up, and six patients with bilateral breast cancer were not included in the lymphedema analysis. Finally, a total of 66 patients were successfully evaluated for the incidence of postoperative lymphedema.

Of the 78 patients, 53 and 36 patients underwent SLNB and ALND, respectively. Of the 36 ALND patients, 11 patients underwent ALND after the SLNB procedure due to a diagnosis of SLN metastasis, and the other 25 patients underwent ALND with no prior SLNB performed. Of these 25 ALND patients, 11 patients also completed neoadjuvant chemotherapy (NAC) due to positive fine-needle aspiration biopsy (FNAB) results.

### ARM node identification

**Fig. 1:**
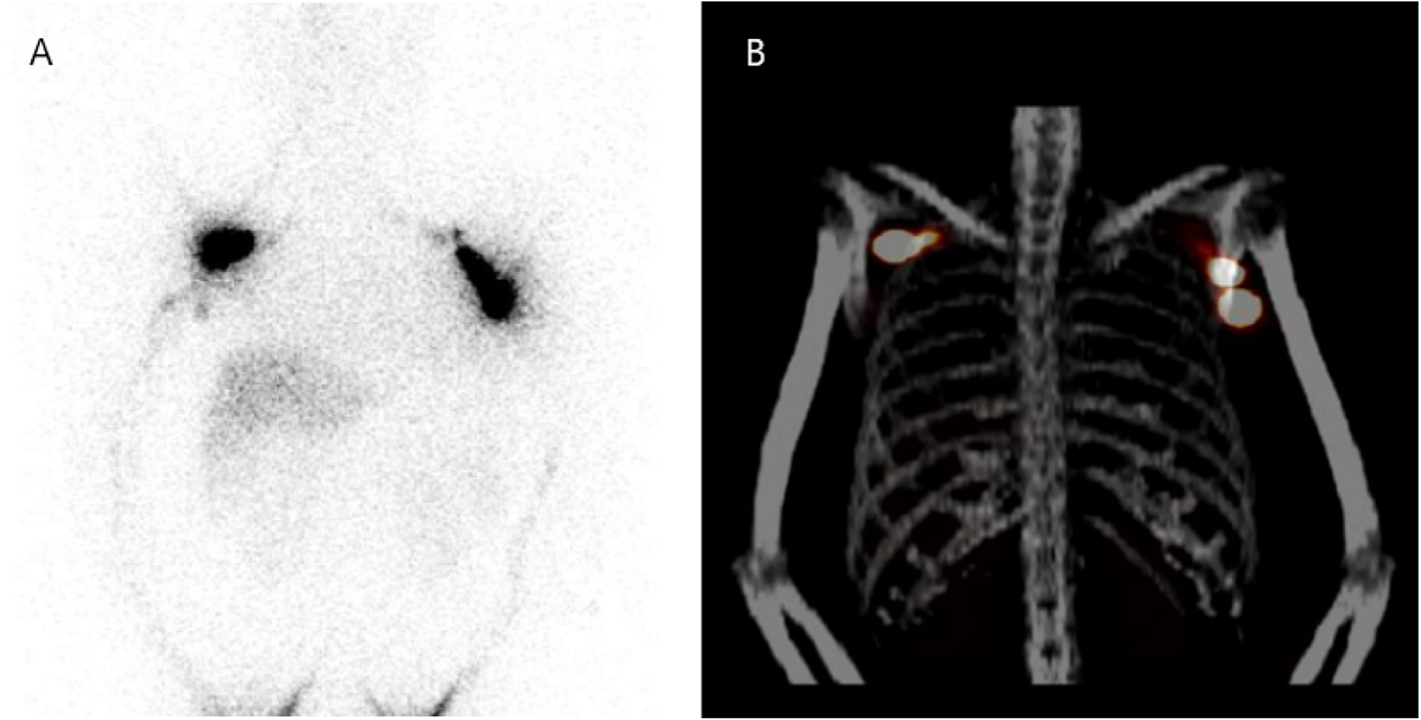
Axillary lymph nodes were visualized with planar scintigraphy (**a**) and SPECT/CT (**b**) imaging

**Fig. 2:**
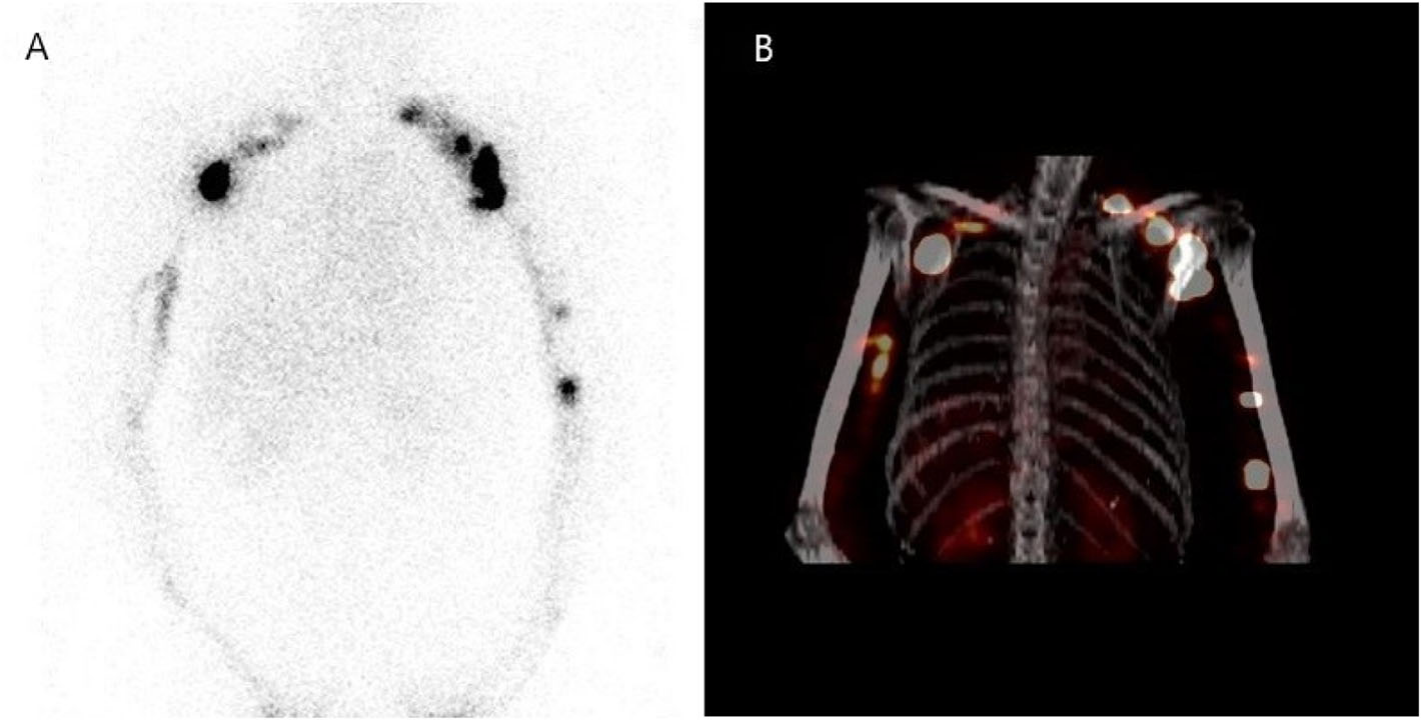
Axillary and clavicular lymph nodes were visualized with planar scintigraphy (**a**) and SPECT/CT (**b**) imaging

The preoperative identification rate of ARM nodes was 100% in all patients with SPECT/CT (Figs. 1 and 2). Of the 53 SLNB patients, all patients’ ARM nodes were identified in the axillary region, and in 22 patients (41.5%), ARM nodes were also identified in the clavicular region. Of the 36 ALND patients, all patients’ ARM nodes were identified in the axillary region, and in 18 patients (50%), ARM nodes were also identified in the clavicular region.

### The oncologic safety of ARM nodes

#### SLNB

Of the 53 patients who underwent SLNB, ARM nodes were removed in 33 (62.3%) patients during the SLNB procedure. The median number of ARM nodes removed was 1 (range, 1–5). Crossover between ARM nodes and breast SLNs was seen in 22 out of 33 patients, and the incidence of ARM node involvement was 1.89% (1/53) in SLNB patients. Another 11 patients had ARM nodes removed, but none had crossover with SLNs, and no positive ARM nodes were found.

Among all 53 patients, SLNs were found to be histologically positive in 15 (28.3%) patients, and secondary ALND was performed in 11 patients.

#### ALND

Of the 36 patients who underwent ALND, ARM nodes were identified in 33 (91.7%) patients. The median number of ARM nodes removed was 2 (range, 1–8). Pathologic results of the ARM nodes were obtained in 30 patients.

Metastatic involvement of the axillary lymph nodes was found in 30 of 36 (83.3%) patients who underwent ALND. The incidence of ARM node involvement was 25% (9/36).

#### Factors associated with ARM node metastatic rates

Among the 30 patients who had ARM node pathologic results in the ALND group, metastatic involvement of the ARM nodes was found in 3 of 9 patients (33.3%) with neoadjuvant chemotherapy (NAC) compared with 6 of 21 patients (28.6%) without NAC (*P* = 1.000). One of 10 patients (10%) who underwent ALND after positive SLNB results had ARM node metastases, while 8 of 20 patients (40%) who underwent only ALND had ARM node metastases (*P* = 0.204). No significant differences were found.

Using univariate analyses, we evaluated the relationship between the incidence of ARM node metastases and the clinicopathological features of the patients, including age, clinical T and N stage, histologic tumour type, oestrogen receptor (ER), progesterone receptor (PR), human epidermal growth factor receptor 2 (Her-2), and Ki-67 (before NAC). No significant differences were found (Table 1). However, there was a positive correlation between the number of positive axillary lymph nodes and ARM node involvement (*P* = 0.036) (Table 2).

**Table 1:** Factors associated with ARM node metastases

Characteristic	ARM positive (*n* = 9)		ARM negative (*n* = 21)		*P* value
	*n*	%	*n*	%	
**Age, years**					1.000
≥ 50	6	66.7	14	66.7	
< 50	3	33.3	7	33.3	
**cT stage**					0.188
T1	1	11.1	8	38.1	
T2	7	77.8	12	57.1	
T3	0	0.0	1	4.8	
T4	1	11.1	0	0	
**cN stage**					0.093
N0	2	22.2	1	4.8	
N1	3	33.3	15	71.4	
N2	2	22.2	4	19.0	
N3	2	22.2	1	4.8	
**Histological type**					0.128
Ductal	6	66.7	8	38.1	
Lobular	1	11.1	0	0	
Mixed	2	22.2	11	52.4	
Unknown	0	0	2	9.5	
**ER status**					0.300
Positive	8	88.9	21	100	
Negative	1	11.1	0	0	
**PR status**					0.418
Positive	4	44.4	14	66.7	
Negative	5	55.6	7	33.3	
**HER2 status**					0.666
Positive	3	33.3	5	23.8	
Negative	6	66.7	16	76.2	
**Ki-67 status**					0.229
Positive	7	77.8	10	47.6	
Negative	2	22.2	11	52.4	
**Neoadjuvant therapy**					1.000
No	6	66.7	15	71.4	
Yes	3	33.3	6	28.6	
**Axillary surgery**					0.204
SLNB+ALND	1	11.1	9	42.6	
ALND	8	88.9	12	57.4	

*cT* clinical tumour, *cN* clinical lymph node, *ER* oestrogen receptor, *PR* progesterone receptor, *Her-2* human epidermal growth factor receptor 2, *SLNB* sentinel lymph node biopsy, *ALND* axillary lymph node dissection, *ARM* axillary reverse mapping

**Table 2:** Correlation between positive axillary lymph nodes and ARM node metastases

Positive axillary lymph nodes	ARM positive (*n* = 9)	ARM negative (*n* = 21)
**Median (1/4 CI, 3/4 CI)**	4 (2.5, 9)	2 (1, 3.5)
**Rank mean**	20.56	13.33
***P*value**	0.036	

*ARM* axillary reverse mapping

### The influence of ARM nodes on BCRL

#### The incidence of lymphedema in the SLNB group compared with the ALND group

Among the 66 patients who were successfully followed up to assess the occurrence of postoperative lymphedema, a total of 12 patients were diagnosed with BCRL by tape-line measurement and had self-reported swelling of the upper limb on the breast cancer-treated side. The incidence of lymphedema in the ALND group was significantly higher than that in the SLNB group (33.3% vs. 5.4%, *P* = 0.003) (Table 3). The incidence of residual ARM nodes was significantly higher in the SLNB group than in the ALND group (67.6% vs. 37.9%, *P* = 0.016) (Table 4).

**Table 3:** The incidence of lymphedema in the SLNB and ALND groups

	SLNB group		ALND group		*P*
	No.	%	No.	%	
**LE+**	2	5.4	10	34.5	0.003
**LE−**	35	94.6	19	65.5	

*LE* lymphedema, *SLNB* sentinel lymph node biopsy, *ALND* axillary lymph node dissection

**Table 4:** The incidence of residual ARM nodes in the SLNB and ALND groups

	SLNB group		ALND group		*P*
	No.	%	No.	%	
**Residual ARM nodes+**	25	67.6	11	37.9	0.016
**Residual ARM nodes−**	12	32.4	18	62.1	

*LE* lymphedema, *SLNB* sentinel lymph node biopsy, *ALND* axillary lymph node dissection, *ARM* axillary reverse mapping

#### SLNB

Of the 37 SLNB patients, lymphedema was identified in 2 (5.4%) patients. ARM nodes were removed in 27 patients, and 2 of those patients developed lymphedema during the follow-up period. ARM nodes were not removed in the remaining 10 patients, and none of those patients developed lymphedema. No significant differences were found between the groups (*P* = 1.000) (Table 5).

**Table 5:** The effect of removing ARM nodes during the SLNB procedure on lymphedema

	ARM nodes removed		ARM nodes not removed		*P*
	No.	%	No.	%	
**LE+**	2	7.4	0	0	1.000
**LE−**	25	92.6	10	100	

*LE* lymphedema, *SLNB* sentinel lymph node biopsy, *ARM* axillary reverse mapping

ARM nodes were not detected in the axillary and/or clavicular regions in 12 patients after the SLNB procedure. Among these patients, ARM nodes were also breast SLNs in 5 patients, and one patient developed lymphedema. In the remaining seven patients, ARM nodes were removed by mistake and did not have any crossover with SLNs in the SLN specimens. After the SLNB procedure, the ARM nodes could still be detected in 25 remaining patients. In five of those patients, the ARM nodes were also breast SLNs. In another eight patients, the ARM nodes were removed by mistake and had no crossover with SLNs in the SLN specimens, and one of the eight patients developed lymphedema. Only SLNs were removed during the SLNB procedure in the remaining 10 patients. The overall incidence of lymphedema was 4% (1/25) in the group with ARM nodes remaining after the SLNB procedure compared with 8.3% (1/12) in the group without any ARM remaining left after the SLNB procedure (*P* = 1.000) (Table 6).

**Table 6:** The effect of retaining ARM nodes after the SLNB procedure on lymphedema

	Without remaining ARM nodes		With remaining ARM nodes		*P*
	No.	%	No.	%	
**LE+**	1	8.3	1	4	1.000
**LE−**	11	91.7	24	96	

*LE* lymphedema, *SLNB* sentinel lymph node biopsy, *ARM* axillary reverse mapping

#### ALND

Of the 29 ALND patients, lymphedema was identified in 10 (34.5%) patients. Eighteen patients had immediate ARM node loss in the axillary and/or clavicular region after ALND. Of the remaining 11 patients, ARM nodes could still be detected in the clavicular or higher axillary region after ALND. The overall incidence of lymphedema was 23.7% in the group with ARM nodes remaining after ALND compared with 38.9% in the other group with no ARM nodes remaining (*P* = 0.694) (Table 7).

**Table 7:** The effect of retaining ARM nodes after the ALND procedure on lymphedema

	Without remaining ARM nodes		With remaining ARM nodes		*P*
	No.	%	No.	%	
**LE+**	7	38.9	3	27.3	0.694
**LE−**	11	61.1	8	72.7	

*LE* lymphedema, *ALND* axillary lymph node dissection, *ARM* axillary reverse mapping

## Discussion

Currently, the mechanisms of lymphedema are complex and there is no consensus. The main cause of lymphedema is possibly due to lymphatic drainage disruption of the upper extremities after axillary surgery. A broad array of lymphedema-associated risk factors have been reported in the published literature, including mastectomy, regional lymph node radiation, ALND, and number of lymph nodes with pathologic involvement [19, 20]. However, until now, no studies could clearly explain why some patients do not develop lymphedema after undergoing ALND, while some patients have lymphedema when only SLNs are removed.

The ARM technique emerged based on a hypothesis that preserving ARM nodes could decrease the occurrence of lymphedema by distinguishing upper limb and breast lymphatic drainage. In 2007, Thompson et al. [4] and Nos et al. [5] reported, for the first time, that preserving ARM nodes during ALND could reduce the incidence of postoperative upper extremity lymphedema.

In our study, we did not deliberately preserve the ARM nodes during axillary surgery. With the help of the ARM technique, we studied the effect of residual ARM nodes on lymphedema after the ALND or SLNB procedure. According to our results, the incidence of lymphedema in the ALND group was significantly higher than that in the SLNB group (33.3% vs. 5.4%, *P* = 0.003). The incidence of residual ARM nodes in the SLNB group was significantly higher than that in the ALND group (67.6% vs. 37.9%, *P* = 0.016), suggesting that more residual ARM nodes in the SLNB group could help decrease the risk of lymphedema. In addition, according to our results, lymphedema was less likely to occur in patients who had ARM nodes remaining after the SLNB or ALND procedure than in patients who did not have ARM nodes remaining after these procedures, which indicates that patients with fewer ARM nodes remaining are possibly at high-risk of developing lymphedema. However, no significant differences were found, which was partly due to the small sample size in this study. To our knowledge, our research was the first to study the relationship between residual axillary ARM nodes and lymphedema after axillary surgery. Our results were consistent with the mechanistic hypothesis of the ARM technique, which proposes that the more ARM nodes remaining after axillary surgery, the less likely LE would occur.

In our study, among the 37 SLNB patients, 15 patients had nonsentinel ARM nodes removed by mistake because they were located near the SLNs, but none of these ARM nodes were positive. Of these patients, only a single patient developed lymphedema, which could have been due to unnecessary ARM node resection. Therefore, during SLNB, avoiding nonsentinel ARM node resection is beneficial.

Previous studies reported that the crossover rates between SLNs and ARM nodes ranged from 2.2 to 28.0% [21, 22]. We observed a higher crossover rate of 41.5% in our surgeries, which could have been because we used a dual mapping technique with methylene and ICG during SLNB. According to the findings of our previous study [23], ICG is sensitive to SLNs, which might result in a greater number of SLNs being removed, and removing more SLNs could lead to the removal of more ARM nodes, leading to lower residual ARM node rates. Therefore, choosing other lymphatic tracer dyes during SLNB could increase the residual ARM node rates.

Theoretically, preserving the lymphatic arm should be able to reduce the occurrence of lymphedema. Many authors have reported the benefit of preserving ARM nodes. However, many problems remain to be resolved in the practical use of the ARM technique. The greatest concern is the risk of leaving positive nodes behind while preserving ARM nodes [24]. In initial studies, Thompson et al. [4], as well as Boneti et al. [12], found no cancer cells in the ARM nodes even when patients had many positive axillary lymph nodes. However, subsequent studies have shown ARM node involvement [16, 25] [26, 27]. Our study reported that the ARM node metastatic rate was 25% (9/36) and 1.89% (1/53) in patients who underwent the ALND and SLNB procedures, respectively. The involvement of ARM nodes was markedly lower in the SLNB group than that in the ALND group, but we are unsure of the reasons behind these findings.

We hope to find factors that can predict the metastatic rate of ARM nodes. Beek et al. [28] showed that patients with clinically positive nodes who received NAC were significantly less likely to have metastatic involvement of the ARM nodes than patients who did not receive NAC (16.5% and 36.8%, respectively). It is thought that neoadjuvant chemotherapy reduces the metastatic burden of disease in the axilla [24]. In our study, however, no significant differences were seen between the incidence of ARM node involvement in patients who received NAC and those who did not receive NAC (33.3% vs. 28.6%, *P* = 1.000). However, only 9 patients who received neoadjuvant therapy were included in this study, so it is difficult to draw a conclusion on the relationship between neoadjuvant therapy and ARM involvement from our research. In addition, no significant differences were found between the incidence of ARM node metastases and clinicopathological features, including age, clinical T and N staging, histological type, ER, PR, Her-2, and Ki-67. However, we did see a positive correlation between the number of positive axillary lymph nodes and ARM node involvement, which was also confirmed by other studies [7]. In fact, it was difficult to distinguish ARM nodes that were positive during surgery and those that were metastatic in the ALND patients. Thus, we do not believe it is feasible to decrease lymphedema by preserving ARM nodes.

However, for patients with SLNB, the ARM technique could help to distinguish nonsentinel ARM nodes and to reduce unnecessary ARM node resection. Moreover, we could use the ARM technique to predict which patients are likely to have a high risk of lymphedema by assessing the rate of residual ARM nodes. Patients with a low residual ARM node rate should undergo early interventions to prevent lymphedema. Our study has some limitations, including the small sample size, single-centre design, and short follow-up times. Larger studies are warranted to verify the results of our study. In addition, due to the diverse diagnostic methods for lymphedema, we used an absolute definition of lymphedema (2 cm) instead of a relative volume difference, and not taking into account the preoperative arm volumes was also a limitation of the design.

## Conclusions

The ARM technique is a good method to help distinguish the upper extremity lymphatic drainage system from that of the breast in the axillary region. The loss of ARM nodes could be correlated with lymphedema after axillary surgery. For patients who undergo SLNB, the ARM technique could assist in more precise SLN resection and avoid unnecessary nonsentinel ARM node resection. For patients who undergo ALND, ARM could help predict which patients are more likely to have a high risk of lymphedema by assessing the rate of residual ARM nodes. Patients with a decreased number of remaining ARM nodes in either the SLNB or ALND procedure are considered to be a high-risk group for lymphedema and should undergo early interventions to prevent lymphedema. However, we do not recommend preserving ARM nodes to prevent lymphedema in patients undergoing ALND because of issues related to oncologic safety issues. Finally, prospective studies are needed to find proper lymphatic tracers in cases where patients undergo ARM and SLNB procedures simultaneously.

## Data Availability

All data generated or analysed during this study are included in this published article and its supplementary information files.

## Abbreviations

BCRL: Breast cancer-related lymphedema
SLNB: Sentinel lymph node biopsy
ALND: Axillary lymph node dissection
SLNs: Sentinel lymph nodes
LE: Lymphedema
ARM: Axillary reverse mapping
ICG: Indocyanine green
SPECT/CT: Single-photon emission computed tomography
FNAB: Fine-needle aspiration biopsy
ER: Oestrogen receptor
PR: Progesterone receptor
Her-2: Human epidermal growth factor receptor 2
